# Integrative Approaches to Ovulation Induction in Polycystic Ovary Syndrome: A Narrative Review of Conventional and Complementary Therapies

**DOI:** 10.3390/biomedicines13112711

**Published:** 2025-11-05

**Authors:** Soo Youn Song

**Affiliations:** Department of Obstetrics & Gynecology, Chungnam National University Sejong Hospital, Chungnam National University School of Medicine, 20, Bodeum 7-ro, Sejong 30099, Republic of Korea; sysong@cnuh.co.kr; Tel.: +82-44-995-3040

**Keywords:** polycystic ovarian syndrome, infertility, ovulation induction

## Abstract

Polycystic ovary syndrome (PCOS) is the most common cause of anovulatory infertility, with ovulation induction remaining the first-line treatment approach. Although letrozole has emerged as the most effective monotherapy, treatment resistance, side effects, and patient preferences have led to increasing interest in adjunctive or alternative approaches. This narrative review summarizes the current evidence for ovulation induction in patients with PCOS, including conventional pharmacologic agents, such as clomiphene citrate, letrozole, gonadotropins, and insulin-sensitizing agents, as well as complementary therapies, such as acupuncture and Chinese herbal medicine. We also examine emerging adjuvants, such as vitamin D, omega-3 fatty acids, sildenafil, and antioxidants that may enhance clinical pregnancy rates or improve endometrial receptivity. While robust evidence supports the use of letrozole as a first-line agent, complementary and integrative therapies may offer additional benefits, particularly in treatment-resistant or preference-driven contexts. Further high-quality studies are needed to clarify the role of combined therapeutic strategies in optimizing fertility outcomes for women with PCOS.

## 1. Introduction

Polycystic ovary syndrome (PCOS) is one of the most common gynecological disorders, affecting an estimated 3–15% of women of reproductive age. The Rotterdam criteria (2003), the most widely accepted criteria for diagnosing PCOS, state that PCOS diagnosis can be made if two of three characteristics are met: clinical or biochemical hyperandrogenism, ovulation abnormalities, and/or polycystic ovaries [1]. The exact pathophysiology of this condition has not been definitively identified, and it can be influenced by environmental pollutants, dietary and lifestyle habits, genetic predisposition, obesity, and gut dysbiosis. An altered gonadotropin-releasing hormone (GnRH) pulse can lead to an imbalance between luteinizing hormone (LH) and follicle-stimulating hormone (FSH) synthesis, and an excess of LH. This excess can stimulate ovarian theca cells to overproduce androgen, disrupt normal follicular growth and prevent ovulation. Hyperinsulinemia, often found in women with PCOS, can reduce sex hormone-binding globulin production, increasing free androgens and affecting ovarian follicle development. Altered ovarian theca activity and granulosa cells, and disrupted folliculogenesis inhibit normal ovulation, and produce the typical polycystic appearance of ovaries. Women with PCOS show various manifestations, such as irregular menstruation, infertility and cardiometabolic complications, including impaired glucose tolerance, obesity, diabetes mellitus, and increased cardiovascular risk [2]. Due to complexity of its pathophysiology, distinguishing between the causes and effects of various symptoms in patients with PCOS is impossible.

As PCOS is the most common cause of anovulation, approximately half of the women with PCOS are infertile. Many medications, including clomiphene citrate and letrozole, are used for ovulation induction in patients with PCOS [3]. However, not all patients respond to these strategies, and concerns over cost, the risk of ovarian hyperstimulation, and multifetal gestation persist. In recent years, interest in complementary and alternative approaches has increased, including acupuncture, Chinese herbal medicine, vitamin supplementation, and antioxidant therapies, either as stand-alone interventions or as adjuncts to conventional treatment (Table 1). Despite the popularity of these methods, especially in certain cultural contexts, evidence remains mixed and often limited by study heterogeneity.

This review aims to provide an integrated synthesis of the current evidence on both conventional and complementary approaches to ovulation induction in women with PCOS. By evaluating recent clinical trials on pharmacologic agents, acupuncture, and Chinese herbal medicine, we seek to highlight not only efficacy but also methodological quality and practical considerations for clinicians with a goal of helping to guide evidence-based decision-making in clinical practice and identify gaps that future studies should address.

## 2. Conventional Ovulation Induction

### 2.1. Clomiphene Citrate

Clomiphene citrate is a selective estrogen receptor modulator [36]. It acts as an estrogen antagonist at the hypothalamus level, depleting the nuclear receptor, resulting in the blockage of negative feedback and increased pulsatile gonadotropin secretions [36,37]. Ovulation is known to occur in 70–80% of patients treated with clomiphene citrate, but the pregnancy rate is as low as 40%, possibly owing to changes in endometrial receptivity and cervical mucus [38,39].

Ovulation induction with clomiphene citrate is started on menstrual cycle days 3–5 and continues for 5 days. The starting day does not seem to affect ovulation outcomes [40]. In the traditional protocol, when the first attempt with 50 mg of clomiphene citrate fails to achieve ovulation, progestin is administered to induce withdrawal bleeding before increasing the clomiphene citrate dose in the next cycle [3]. Recently, the stair-step method, in which clomiphene citrate levels are increased within the same cycle without progestin withdrawal bleeding, has been widely adopted for ovulation induction [41]. The stair-step method decreases the time to ovulate compared with the traditional protocol [41,42,43,44], and some studies have reported increased ovulation rates [41,44,45].

Side effects of clomiphene citrate are uncommon; however, some patients experience vasomotor symptoms, adnexal tenderness, nausea, or headache after taking the pill [46]. Ovulation induction with clomiphene citrate is also associated with a higher risk of multifetal pregnancies [47,48,49].

### 2.2. Letrozole

Letrozole inhibits aromatase, a rate-limiting enzyme in estrogen production. It blocks negative feedback in the hypothalamus-pituitary axis, thus increasing the pulsatile secretion of gonadotropin [50,51]. was later used for ovulation induction in patients who did not respond to clomiphene citrate [52]. Similar to clomiphene citrate, letrozole is administered for 5 days starting from menstrual cycle days 3–5. According to a recent retrospective study, the administration of letrozole on day 5 resulted in higher ovulation and conception rates than on day 3 [53]. An extended regimen in which the duration of letrozole administration was increased from 5 to 10 days resulted in 65.7–92.75% successful ovulations in patients who did not respond to 5 days of treatment [54].

Based on clinical data on the efficacy of letrozole with that of clomiphene citrate, there are several meta-analyses have compared the fertility outcomes of letrozole and clomiphene citrate in patients with PCOS. A meta-analysis conducted in 2011 found no significant differences in pregnancy rates between letrozole and clomiphene citrate [55]. Two 2015 meta-analyses by Misso found that letrozole resulted in higher live birth and pregnancy rates, although the ovulation rate did not differ between the two groups [55,56]. Since then, several meta-analyses have reported not only higher ovulation rates but also higher pregnancy and live birth rates with letrozole than with clomiphene citrate [57,58,59]. The most recent review published in 2024 evaluated 50 randomized controlled studies enrolling 75,009 women and concluded that letrozole treatment resulted in a thicker endometrium and higher ovulation and pregnancy rates in patients with PCOS compared to those treated with clomiphene citrate [60].

Studies comparing letrozole and clomiphene using the stair-step method have shown similar results. Thomas et al. reported that the stair-step method with letrozole reduced the time to ovulation compared to the stair-step method with clomiphene citrate, although no differences in ovulation or pregnancy rates were found between the two groups [61]. In another study, the stair-step method using letrozole resulted in not only a shorter time to ovulation but also higher ovulation and pregnancy rates [62]. A recent study showed a better ovulation rate with the stair-step method using letrozole compared to letrozole alone, but comparable pregnancy rates [63]. Based on the current data, letrozole appears to be a better first-line treatment for ovulation induction in patients with PCOS, regardless of the protocol used.

### 2.3. Gonadotropin

Gonadotropins, including human menopausal gonadotropin or recombinant FSH, can be used to induce ovulation in patients with hypogonadotropic hypogonadism and eugonadotropic anovulation, such as those with PCOS. A recent meta-analysis showed no differences in clinical pregnancy, miscarriage, multiple pregnancies, or live birth rates between different types of exogenous gonadotropin injections [64]. Exogenous gonadotropins can be used for ovulation and achieve successful pregnancies in many patients. One study showed a cumulative pregnancy rate of 90% and a live birth rate of 85% after ovulation induction with exogenous gonadotropin [65]. However, gonadotropin is seldom considered a first-line treatment for ovulation induction owing to the higher risk of multifetal pregnancies, ovarian hyperstimulation syndrome, and spontaneous miscarriage [66,67]. However, gonadotropins resulted in a higher live birth rate in patients who failed to achieve a successful pregnancy after an initial attempt with clomiphene citrate than continuous treatment with clomiphene citrate [68].

### 2.4. Combination Therapy

As clomiphene citrate and letrozole have different mechanisms of ovulation induction, many studies have investigated combination therapy using both letrozole and clomiphene citrate compared to letrozole alone, but with conflicting results. A randomized controlled study in 2019 did not find any differences in endometrial thickness, ovulation rates, or clinical pregnancy rates between patients given combination therapy and letrozole alone [69]. Similarly, a retrospective study in 2023 did not find a significant difference in ovulation rates between the groups [70]. Conversely, two recent meta-analyses of RCTs have found that combination therapy resulted in a higher ovulation rate than letrozole [71,72].

However, the biggest drawback of using exogenous gonadotropins for ovulation induction is the risk of ovarian hyperstimulation syndrome and multifetal pregnancies because polycystic ovaries can be more sensitive to gonadotropins [66,67]. Combining gonadotropin with other agents used for ovulation induction has been suggested as a novel method to increase the pregnancy rate and reduce the complications of gonadotropin [73]. In a randomized controlled study published in 2023, dominant follicle formation was confirmed in 82.9% of patients who failed to ovulate after clomiphene citrate or letrozole treatment [74]. A retrospective study conducted in 2023 compared three ovulation induction methods: gonadotropin, letrozole with gonadotropin, and clomiphene citrate with gonadotropin. The combination of letrozole and gonadotropin was superior in terms of ovulation and clinical pregnancy rates in the patients [75]. Consistent with these results, a randomized controlled study published in 2023 showed that sequential treatment with letrozole and gonadotropins was superior to letrozole alone in terms of ovulation induction and pregnancy rates [76].

### 2.5. Tamoxifen

Tamoxifen is a selective estrogen receptor modulator. Tamoxifen acts as an estrogen receptor agonist on the endometrium and vaginal mucus compared with clomiphene citrate, which has an anti-estrogenic effect on the endometrium and cervical mucus [77]. Many studies comparing tamoxifen with clomiphene citrate have shown conflicting results. Most studies showed favorable results of tamoxifen on endometrial thickness compared to clomiphene citrate [78,79,80]; however, one study reported unfavorable results [81]. Regarding pregnancy outcomes, some studies showed comparable pregnancy rates [81,82,83], whereas others showed better results with clomiphene citrate [84] or tamoxifen compared to the others [85,86]. A 2018 meta-analysis of randomized clinical trials found that tamoxifen and clomiphene citrate were comparable; however, a subgroup analysis with case–control studies favored tamoxifen in terms of ovulation and pregnancy rates [87].

The ovulation rate in patients with clomiphene citrate-resistant PCOS was better with letrozole than with tamoxifen; however, the pregnancy rate was not significantly different [88].

## 3. Adjuncts to Conventional Ovulation Induction

### 3.1. Insulin-Sensitizing Agents

Metformin is an insulin-sensitizing drug that can reduce insulin resistance, which is common in patients with PCOS, and thus enhances fertility [89,90,91]. However, metformin alone may not be sufficient to induce ovulation. Tang et al. compared metformin alone with placebo and found that metformin did not enhance ovulation or pregnancy rates [92].

Metformin showed positive effects when combined with other agents used for ovulation induction. A meta-analysis in 2015 showed that metformin combined with clomiphene citrate resulted in ovulation and pregnancy rates similar to letrozole [93]. Another meta-analysis in 2019 showed improved ovulation and clinical pregnancy rates with combination therapy compared with clomiphene citrate alone, but the live birth rate was not different [94]. Similarly, a meta-analysis in 2022 showed that supplementing metformin with clomiphene citrate improved clinical pregnancy rates but not ovulation or live birth rates [95]. Conversely, the most recent double-blind randomized controlled trial showed that combination therapy was not superior to clomiphene citrate alone in terms of ovulation, clinical pregnancy, or live birth rates [96].

The combination of metformin with gonadotropin was shown to enhance live birth rates in two meta-analyses [97,98].

Pioglitazone is a drug that affects peripheral insulin sensitivity. Pioglitazone affects the outcome of ovarian stimulation by influencing ovarian stromal blood flow [99]. However, most studies have failed to demonstrate the effects of pioglitazone on ovulation induction [100,101].

Myo-inositol is an insulin sensitizer that can improve hormone profiles and enhance metabolic factors in patients with PCOS. Regarding ovulation induction, a 2017 study showed an improved clinical pregnancy rate when inositol was administered with gonadotropins [102]. Similarly, a 2019 study showed better live birth rates when inositol was administered with metformin than metformin alone [103].

### 3.2. Dexamethasone

Hyperandrogenism affects approximately 60–80% of patients with PCOS and can cause ovulatory dysfunction and fertility [104]. Glucocorticoids can decrease adrenal androgen synthesis, thus reducing hyperandrogenic anovulation and controlling the release of gonadotropins by affecting the pulsatility of gonadotropin-releasing hormone [105,106]. A randomized controlled study showed that adding dexamethasone to letrozole increased pregnancy rates in patients with PCOS [107]. Another study showed that treating letrozole-resistant PCOS patients with letrozole plus dexamethasone resulted in a 79% ovulation rate and a live birth rate similar to that of the control group, which was not resistant to letrozole [108]. Similarly, administering glucocorticoids to clomiphene-resistant PCOS patients improved ovulation and pregnancy rates, regardless of whether they had high [109,110] or normal dehydroepiandrosterone sulfate levels [31,111,112].

### 3.3. Dopamine Agonist

Cabergoline is an ergot-derived dopamine agonist used as a prolactin inhibitor. It acts on dopamine receptors and LH and prolactin levels due to insufficient dopamine agonists [113,114]. Cabergoline combined with clomiphene citrate showed a significant increase in ovulation and pregnancy rates compared to clomiphene alone, even in PCOS patients with normal prolactin levels [115,116,117].

Bromocriptine, another dopamine agonist, does not improve ovulation or pregnancy rates in PCOS patients who are resistant to clomiphene citrate and have normal prolactin levels [35,114].

### 3.4. Aspirin

Aspirin has anti-inflammatory, antipyretic, and analgesic effects [118,119]. Aspirin can enhance uterine blood flow in patients with PCOS, which can induce endometrial development by improving local tissue microcirculation [120] and enhance blood circulation in the uterus and ovaries, thus improving ovulation [120]. This may prevent the expulsion of the embryo by reducing the excitability and sensitivity of the uterine muscle [121]. Based on these possible mechanisms, a recent meta-analysis of 10 randomized controlled trials showed that aspirin could increase endometrial thickness, ovulation rates, and pregnancy rates and reduce abortion rates [121]. However, the sample size of most studies was very small, and the studies were not standardized, so no definitive conclusions could be drawn.

### 3.5. Sildenafil

Women treated with clomiphene citrate showed decreased numbers and diameters of the endometrial glands and slow glandular maturation [122]. Vasodilators, such as sildenafil, can be used to increase blood flow and endometrial thickening [123]. Sildenafil citrate inhibits phosphodiesterase 5 and cGMP-dependent protein kinases, maximizing the effect of nitric oxide, and leading to smooth muscle cell relaxation [124]. In a randomized controlled study in 2015, sildenafil citrate combined with clomiphene citrate increased endometrial thickness and pregnancy rates [125]. Another randomized controlled study showed that PCOS patients who failed to conceive after clomiphene citrate treatment could benefit from the addition of sildenafil citrate because it increases endometrial thickness and clinical pregnancy rates [126]. The most recent study conducted in 2022 also showed improved endometrial thickness, ovulation rates, and clinical pregnancy rates in patients with PCOS treated with combination therapy compared to clomiphene citrate alone [127]. A recent meta-analysis showed that infertile patients with a thin endometrium could benefit from vasodilators such as sildenafil citrate [128,129], so adding sildenafil citrate to PCOS patients undergoing ovulation induction with clomiphene citrate might be a good option to improve clinical pregnancy rates. A randomized comparative study by Mohammed et al. showed adding sildenafil citrate to letrozole improved endometrial thickness and clinical pregnancy rates in patients with PCOS [130].

### 3.6. Vitamin D

The role of vitamin D in the pathogenesis of PCOS has increased, since approximately 67–85% of patients with PCOS are deficient in vitamin D [131]. One possible mechanism underlying the role of vitamin D in PCOS-associated infertility is that it can prevent oxidative stress and maintain calcium ion levels to maintain the resting potential of cells, thus improving fertilization, embryo development, and implantation [132]. A recent meta-analysis analyzed 20 randomized controlled studies with conflicting results and concluded that vitamin D improved ovulation and clinical pregnancy rates but not cumulative pregnancy rates [133]. Most recently, a randomized controlled study showed that 30,000 IU of vitamin D supplemented with calcium for 12 weeks significantly increased the ovulation rate [134]. A randomized double-blind controlled trial on the effect of vitamin D supplementation is ongoing to assess the ovulation rate of women with PCOS [135].

### 3.7. Omega 3

A diet rich in omega-3 polyunsaturated fatty acids (w3 PUFAs) has been shown to affect the quality of oocytes and implantation, thus positively impacting fertility [136]. Omega-3 fatty acid intake is associated with higher fecundability [137,138,139]. Omega-3 fatty acid intake improves the metabolic status and hormone levels in patients with PCOS [140,141]. According to a randomized controlled study in 2023, ω3 FA supplementation combined with clomiphene citrate increased clinical pregnancy rates compared with clomiphene citrate supplementation alone [142]. It also reduced endometrial thickening in overweight and obese patients [142].

### 3.8. Coenzyme Q10

Coenzyme Q10 is an antioxidant supplement that neutralizes free radicals and reduces oxidative stress. One study in PCOS patients reported improved ovulation induction and clinical pregnancy rates compared to clomiphene citrate alone [143]. However, another study found no differences in ovulation rates between the two groups [6].

## 4. Complementary and Traditional Therapies

### 4.1. Acupuncture

Acupuncture has been extensively investigated as a complementary therapy for ovulation induction in patients with PCOS [144]. The proposed mechanisms include the modulation of neuroendocrine function, improvements in insulin sensitivity, and the enhancement of ovarian blood flow [145,146,147]. Electroacupuncture, a technique that combines traditional acupuncture with electrical stimulation, has garnered attention for its potential to further enhance therapeutic effects [145]. Preclinical studies suggest that electroacupuncture may improve oocyte quality and embryonic development by upregulating the IRS-1/PI3K/GLUT4 signaling pathway, downregulating anti-Müllerian hormone overexpression, and increasing P450 aromatase expression [148]. It may also reduce granulosa cell autophagy by inhibiting the PI3K/AKT/mTOR pathway by downregulating LncMEG3 expression [149].

Evidence comparing acupuncture with conventional medical therapies is mixed (Table 2). A 2014 meta-analysis of 31 articles showed that acupuncture may be effective for PCOS patients, and a 2019 network meta-analysis of 39 randomized controlled trials (RCTs) reported that acupuncture was superior to Western medications for ovulation induction and pregnancy rates in PCOS patients [150]. However, a 2020 meta-analysis of 22 studies found no significant difference in ovulation or live birth rates between acupuncture and standard treatments [151]. More recently, a 2023 meta-analysis of six RCTs found that acupuncture combined with moxibustion produced ovulation rates comparable to clomiphene citrate [152].

Acupuncture has also been studied as an adjunct to conventional ovulation induction. A 2022 meta-analysis of nine RCTs comparing metformin plus acupuncture versus metformin alone demonstrated improved ovulation and pregnancy rates in the combination group [153].

Despite promising individual studies, the overall evidence regarding the efficacy of acupuncture remains inconclusive. A 2016 Cochrane review based on five RCTs suggested that acupuncture may increase ovulation frequency, but emphasized the low quality of evidence due to methodological limitations [154]. Similarly, a 2017 meta-analysis found limited support for acupuncture in improving ovulation and pregnancy outcomes [155]. Likewise, both a 2023 umbrella review of systematic reviews and a narrative review concluded that while acupuncture is widely used in clinical practice, the current evidence is insufficient and inconsistent to establish its efficacy with confidence [146,156]. More recently, a network meta-analysis in 2024 suggested the role of acupuncture as a complementary treatment to clomiphene citrate and letrozole in improving pregnancy rates [157]. Likewise, an umbrella review of systematic reviews and meta-analyses of 38 meta-analyses showed that acupuncture therapies were significantly associated with higher ovulation and pregnancy rates [158]. An upcoming multicenter randomized trial of acupuncture and letrozole on live birth in infertile women with PCOS plans to recruit 1100 women from 28 hospitals and hopefully add valuable findings to the existing evidence [159].

Even though some studies showed promising results with acupuncture, evidence regarding its ability to induce ovulation in PCOS patients is limited due to the poor quality and small sample size of the studies, as well as their potential biases and heterogeneous results. GRADE criteria suggest that the certainty of evidence for acupuncture for ovulation induction in PCOS patients can be considered low to moderate overall, primarily due to the risks of bias, imprecision, and inconsistency. Further well-designed, large-scale studies are needed to clarify the role of acupuncture, determine the optimal treatment protocols, and assess long-term outcomes related to ovulation induction in women with PCOS.

**Table 2:** Meta-analyses and systematic reviews on acupuncture for ovulation in patients with polycystic ovarian syndrome.

Author, Year	Type of Study	Treatment	Included Studies	Main Finding	Overlapping Articles	Bias Ranking	Level of Evidence
Bai, 2024 [158]	Umbrella review	Acupuncture	20 meta-analyses	Acupuncture therapies were significantly associated with a higher ovulation rate (RR 1.25, 95% CI 1.15–1.35)	8 articles overlap with [146]	NA	
Deng, 2024 [160]	Network meta-analysis	Acupuncture as a combination therapy	28 RCTs	Ear point pressure + herbal enema + herbal, acupuncture and moxibustion + herbal, fire acupuncture + herbal, and acupuncture + herbal improved ovulation rates better than acupuncture, herbal, and Western medicine monotherapy	4 articles with [161], 1 with [154]	High ~ unclear risk	
Yang, 2023 [152]	Meta-analysis	Acupuncture and moxibustion combined with clomiphene	6 RCTs	Acupuncture and moxibustion was effective in improving ovulation-promoting effects and pregnancy outcomes in PCOS patients. The ovulation-promoting effect of acupuncture and moxibustion, or combined with clomiphene, was similar to that of clomiphene alone, but acupuncture & moxibustion combined with clomiphene had more advantages in improving pregnancy rates	1 article with [151], 1 article with [154], and 1 article with [161]	Mostly low risk	Low
Yang, 2023 [146]	Overview of systematic reviews	Acupuncture	11 systematic reviews	Combining acupuncture with other medicines effectively improved clinical pregnancy and ovulation rates. When compared with medicine alone, acupuncture alone also improved clinical pregnancy rates. The efficacy and safety of acupuncture for PCOS remain uncertain due to the limitations and inconsistencies of the current evidence	-	NA	
Chen 2022 [153]	Meta-analysis	Acupuncture combined with metformin vs. metformin	9 RCTs	Compared with metformin alone, acupuncture combined with metformin had a positive effect on ovulation rates in PCOS patients (RR 1.18, 95% CI 1.08–1.29, *p* = 0.57, I^2^ = 0%)	2 articles overlap with [161]	High risk	Low
Li 2022 [161]	Meta-analysis	Acupuncture combined with moxibustion	25 RCTs	Acupuncture combined with moxibustion as a complementary therapy to basic treatments improved ovulation (RR 1.31, 95% CI 1.22–1.40, *p* < 0.01)	-	Moderate ~ high risk	Low
Wu 2020 [151]	Meta-analysis	Acupuncture	22 RCTs	Significantly high rate of ovulation with acupuncture (RR 1.11, 95% CI 1.00–1.24, I^2^ = 65%; *p* = 0.05)	4 articles overlap with [154]	High ~ unclear risk	Very low ~ low
Song 2019 [150]	Network meta-analysis	Acupuncture	39 RCTs	Ovulation rates were better for acupuncture-medication therapy than for Western medication	-	NA	
Lim 2019 [154]	Meta-analysis	Acupuncture	8 RCTs	For true acupuncture versus sham acupuncture, the authors could not exclude clinically relevant differences in ovulation rates (SMD 0.02, 95% CI—0.15–0.19, I^2^ = 0%)	5 articles overlap with [154]	High risk	Low ~ moderate
Jo 2017 [155]	Meta-analysis	Acupuncture	3 RCTs	Acupuncture was more likely to improve ovulation rates compared with no acupuncture (MD 0.35, 95% CI: 0.14–0.56)	1 article overlaps with [154]	High ~ unclear risk	Low
Lim 2016 [154]	Meta-analysis	Acupuncture	5 RCTs	Insufficient evidence to support the use of acupuncture to treat ovulation disorders in women with PCOS (MD—0.03, 95% CI—0.14–0.08)	-	High ~ unclear risk	Low

PCOS, polycystic ovarian syndrome; RCT, randomized controlled trial; NA, not applicable; RR, relative risk; CI, confidence interval; SMD, standardized mean difference; MD, mean difference.

### 4.2. Chinese Herbal Medicine

Traditional Chinese herbal medicine (CHM) has a long history of use in treating menstrual irregularities and infertility. Many CHM compounds have been shown to exert potential therapeutic effects on gynecologic conditions through various mechanisms (Table 3). These include activation of the PI3K/Akt signaling pathway, which supports reproductive cell function, and inhibition of the NF-κB pathway, which reduces inflammation in neural and reproductive tissues [162,163,164]. CHM may also suppress the production of pro-inflammatory cytokines, such as tumor necrosis factor (TNF)-α, interleukin (IL)-1β, and IL-6, and activate SIRT1/STAT3 and Keap1/Nrf2/ARE signaling pathways, enhancing cellular defenses against oxidative stress in reproductive tissues [165,166,167]. In the context of PCOS, CHM formulations have been proposed to improve hormonal balance, reduce insulin resistance, and support ovulation [168]. However, clinical evidence on their efficacy in inducing ovulation is inconsistent (Table 4).

A 2010 meta-analysis of four RCTs found that the addition of CHM to clomiphene citrate improved pregnancy rates in PCOS patients. However, no significant benefit was found when CHM was directly compared to clomiphene citrate or laparoscopic ovarian drilling [169]. Similarly, a 2018 meta-analysis by Kwon et al. suggested that herbal medicine combined with moxibustion may enhance pregnancy rates, either as a stand-alone treatment or adjunctive therapy. However, the included studies were of poor methodological quality [170].

In contrast, a 2017 review by Arentz et al. concluded that no high-quality evidence supported the effectiveness of herbal medicine or nutritional supplements for women with PCOS [171]. Likewise, Zhou et al. conducted meta-analyses in 2016 and 2021, both of which found insufficient evidence to support CHM in improving fertility outcomes in patients with PCOS [169]. A 2021 overview of systematic reviews evaluating CHM for PCOS also concluded that while some studies reported higher ovulation rates with combination therapy compared to conventional ovulation-inducing medicine alone, the overall evidence did not support CHM as more effective than conventional treatments [172]. More recently, a 2024 network meta-analysis of 28 RCTs reported that combination therapy involving Chinese herbal medicine was more effective for infertile PCOS patients than acupuncture, CHM monotherapy, or conventional medication alone [160].

Like the above-mentioned meta-analysis in 2018, several studies have been published on other types of traditional methods, such as wet/dry cupping and moxibustion, as treatments for PCOS. Reports on cupping suggested possible effects on menstrual cycle regulation, but no report has been published on ovulation induction. A few studies suggested that moxibustion could reduce blood glucose and testosterone levels and may have positive effect on fertility [160,173]. Other herbal remedies are also commonly used to treat PCOS [174]. Herbs, such as celery, white bryony, St John’s wort, sage, and chickpea, can improve metabolic parameters, such as the lipid profile, serum glucose levels, and insulin resistance [175]. Celery and St John’s wort, as well as fennel, anise, saffron, horehound, and mallow, can also restore hormonal balances [176]. Some herbs, such as anise, St John’s wort, saffron, and sage, can decrease inflammatory markers, such as TNF-α and IL-6 [177]. Improvements in ovarian histology, dominant follicle formation, and ovulation were shown with watercress, chamomile, Bermuda grass, and mistletoe [174].

As with the acupuncture studies, although several studies have suggested the potential benefits of CHM for ovulation induction in women with PCOS, the overall quality of evidence remains limited. Most RCTs have been conducted in single centers with relatively small sample sizes, reducing the generalizability of their findings. Moreover, methodological quality varies considerably, with frequent issues related to unclear randomization procedures, the lack of allocation concealment, and limited blinding, which is particularly challenging in acupuncture studies. Also, considerable heterogeneity in treatment protocols exists; herbal formulations and outcome definitions further complicate comparisons across studies. The certainty of evidence for acupuncture and CHM-based interventions based on GRADE criteria can be considered low overall. Further well-designed, large-scale studies are needed to clarify the role of CHM for ovulation induction in women with PCOS.

**Table 3:** Active ingredients in Chinese herbal medicine and their mechanisms.

Phytoconstituents	Mechanism	Efficacy in PCOS Patients	References
Quercetin	Inhibits PI3K/Akt/FoxO3a pathway Enhances hypothalamic-pituitary-ovarian axis Lowers hyperandrogenism Decreases LH/FSH ratio, enhances CYP19A1 and CYP11A1 expression Improves insulin resistance in PCOS rats and restores the estrous cycle of rats	Modulates serum FSH and AMH Decreases testosterone levels Improves pregnancy rates	[178,179]
Cinnamon	Enhances insulin sensitivity Increases serum FSH, IGFBP-1, and decreases insulin, IGF-1	Improves menstrual cycles and ovarian size in patients Beneficial effects on metabolism	[180]
Curcumin	Upregulates the expression of the peroxisome proliferator-activated receptor γ (PPAR-γ) gene, the peroxisome proliferator-activated receptor γ coactivator 1α (PGC1α) gene, the low-density lipoprotein receptor gene, and the activity of glutathione peroxidase	Reduces body mass index, fasting blood glucose, insulin levels, and the degree of insulin resistance Reduces complications related to oxidative stress in PCOS patients and improves insulin sensitivity	[181]
Berberine	Relieves the progression of PCOS by affecting the production of short-chain fatty acids by intestinal flora in PCOS mice Neuroprotective and cardiovascular protective effects in an animal model Improves insulin resistance, cell viability, and inhibits apoptosis in granulosa cells	Lipid-lowering and insulin resistance-improving effects alleviate insulin resistance, improve glucose and lipid metabolism and reproductive endocrine status	[182]
Apigenin	Inhibits antioxidant effects and downregulates TNF-α and IL-6 expression	Improves ovarian histologic alteration and follicular dynamics	[183,184]
Resveratrol	Improves estradiol, LH, and testosterone Reduces mTOR and Akt expression Improves insulin resistance and glycolysis by activating SIRT2	Improves menstrual cycles	[185]
Genistein	Modulates PCOS symptoms through the ER-Nrf2-Foxo1-ROS pathway	May enhance insulin sensitivity and has potential to regularize menstrual cycles	[186]
Licorice	Inhibits 11 beta hydroxysteroid dehydrogenase type 2 enzyme	Helps maintain male hormone levels	[187]
Lignans	Upregulates PPAR-r gene and anti-inflammatory and antioxidant function	Helps regulate blood sugar, promote weight loss, and suppress male hormone levels	[187]
Catechins (L-epicatechin)	Decreases insulin resistance, LH/FSH ratio, and inflammatory cytokines	Reduces hormone levels associated with ovarian cysts and promotes weight loss	[188]
Ginseng	Reduces plasma LH level and improve endocrine status	Lowers blood sugar levels	[187]

PCOS, polycystic ovarian syndrome; PI3K/Akt/FoxO3a, Phosphoinositide 3-kinas/protein kinase B/forkhead box protein O 3a; FSH, follicle stimulating hormone; LH, Luteinizing hormone; AMH, Anti mullerian hormone; CYP, Cytochrome P450 enzyme; IGFBP, Insulin-like growth factor-binding protein; IGF, Insulin-like growth factor; TNF, Tumor necrosis factor; IL, Interleukin; mTOR, Mammalian target of rapamycin; SIRT2, Sirtuin 1; ER-Nrf2-Foxo1-ROS, Estrogen receptor—nuclear factor erythroid 2-related factor 2-forkhead box protein O1-reactive oxygen species; PPAR, Peroxisome proliferator-activated receptor.

**Table 4:** Meta-analyses and systematic reviews on Chinese herbal medicine for ovulation in patients with polycystic ovarian syndrome.

Author, Year	Type of Study	Treatment	Included studies	Main Finding	Overlapping Articles	Bias Ranking	Level of Evidence
Deng, 2024 [160]	Network meta-analysis	Combined CHM	28 RCTs	Moxibustion + herbal, fire acupuncture + herbal, acupuncture + herbal, electroacupuncture + herbal, and acupoint application + herbal improved clinical pregnancy rates better than acupuncture, herbal, and Western medicine monotherapy	4 articles overlap with [170], 1 article overlaps with [189], 1 article overlaps with [190]	High ~ unclear risk	
Rong 2023 [190]	Meta-analysis	Add-on of the Guizhi Fuling formula	16 RCTs	The Guizhi Fuling formula plus Western medicine significantly improved ovulation and pregnancy rates better than Western medicine alone. Ovulation rate (RR 1.24, 95% CI 1.15–1.34)	-	Unclear	Low
Zhou 2023 [189]	Meta-analysis	Xiao Yao San	9 studies	Xiao Yao San plus conventional medicines for PCOS significantly improved ovulation rate and pregnancy rate Ovulation rate: intervention vs. control (OR 2.45, 95% CI 1.94 to 3.08, *p* < 0.001)	1 article overlaps with [170]	Moderate ~ high	Low
Tang 2021 [191]	Meta-analysis	Kuntai capsule combined with letrozole	17 studies	Combination group showed improved ovulation and pregnancy rates compared to the letrozole group Ovulation rate: combination vs. letrozole (OR 3.36, 95% CI 1.90–5.94, *p* < 0.0001)	1 article overlaps with [170]	High	Low
Wang 2021 [172]	Overview of systematic reviews	CHM	18 studies	There is insufficient evidence to suggest that improved efficacy is achieved by the combined use of Chinese and Western medicine compared with Western medicine alone in treating PCOS	NA	High	Low
Zhou 2021 [169]	Meta-analysis	CHM	8 RCTs	There is insufficient evidence to support the use of CHM for subfertile women with PCOS. Pregnancy rate CHM vs. Clomiphene (OR 1.41, 95% CI 0.63 to 3.19) CHM + clomiphene vs. clomiphene (OR 3.06, 95% CI 2.05 to 4.55)	5 articles overlap with [169]	Unclear	Very low ~ low
Kwon 2018 [170]	Meta-analysis	CHM combined with moxibustion	9 RCTs and quasi-RCTs	CHM combined with moxibustion might be beneficial for treating PCOS, but similar ovulation rate: Oriental herbal medicine + moxibustion vs. Western medicine (RR 1.23, 95% CI 0.98–1.55)	-	High ~ unclear	Low
Ma 2017 [192]	Meta-analysis	CM with letrozole	8 RCTs	Cycle ovulation rate in groups co-treated with CHM and letrozole was higher than in groups receiving letrozole monotherapy (OR 2.28, 95% CI 1.58–3.30, *p* < 0.0001)	-	Unclear	Low
Zhou 2016 [169]	Meta-analysis	CHM	5 RCTs	There is insufficient evidence to support the use of CHM for women with PCOS and subfertility. Pregnancy rate CHM vs. clomiphene (OR 1.98, 95% CI 0.78–5.06) CHM + clomiphene vs. clomiphene (OR 2.62, 95% CI 1.65–4.14)	4 articles overlap with [169]	Unclear	Very low
Reid 2015 [193]	Meta-analysis	CHM	40 RCTs	18% increased chance of improved ovulation with CHM compared to standard WM therapy in women with previously anovulatory cycles (RR 1.18, 95% CI 1.12–1.25, *p* < 0.001)	5 articles overlap with [169]	High	Low
Zhang 2010 [169]	Meta-analysis	CHM	4 RCTs	No evidence of statistically significant difference in improving ovulation between CHM and clomiphene or between CHM plus laparoscopic ovarian drilling	NA		

CHM, Chinese herbal medicine; PCOS, polycystic ovarian syndrome; RCT, randomized controlled tria; RR, Relative risk; OR, Odds ratio; CI, Confidence interval.

Some considerations should be made before adding adjuncts to traditional ovulation induction methods. Metformin is commonly associated with gastrointestinal problems, such as abdominal pain or discomfort, nausea, diarrhea, and vomiting. One study found that 12 patients discontinued treatment due to abdominal pain and diarrhea [95]. Dexamethasone was associated with vertigo, vomiting, and a meta-analysis showed that adding dexamethasone to clomiphene did not significantly increase the incidence of vertigo or vomiting [194]. Aspirin use is accompanied by concerns for possible bleeding and gastrointestinal side effects, but no statistically significant difference in the incidence of adverse reactions was seen between the two groups in a previous study [121]. Sildenafil may be associated with headache, tachycardia, and hypotension, but the symptoms were reported to be mild [127]. Neither vitamin D nor omega 3 was associated with significant side effects [133]. Acupuncture was associated with localized side effects, such as back muscle spasms and bruising. Other types of side effects included diarrhea, liver dysfunction, and edema. The obstetric complications reported in true acupuncture groups included threatened abortion, ectopic pregnancy, preterm labor, gestational diabetes, and pre-eclampsia [154]. Regarding CHM, gastrointestinal symptoms, such as bloating, nausea, loose stools, and vomiting, have been reported. Other studies reported tiredness, skin breakout, ovulation pain, light-headedness, and headache [195]. Other symptoms included flu-like symptoms, such as lethargy, joint pain, and abnormal uterine bleeding [196]. Long-term use of phytoestrogens such as genistein may disrupt the pituitary-hypothalamus axis resulting in long-term inhibition of endogenous steroid production, and may worsen hypertension due to their vasopressin activity [197]. Some herbs may interact with other mediciations. For example, berberine has been documented to have interactions via cytochrome P450 enzyme inhibition and glucose-lowering potential, raising concern for use with antidiabetic and anticoagulant drugs [198]. Cinnamon has been associated with acute hepatitis [199]. Licorice produces a pseudo-aldosteronism syndrome and is considered contraindicated or high-risk in uncontrolled hypertension and pregnancy, and black cohosh has been implicated in liver injury [200]. A meta-analysis found no significant increase in serious adverse events such as liver or kidney impairment or allergies. However, the safety of CHM for women with subfertile PCOS remains unclear [169].

Although many RCTs report only mild, tolerable adverse events or make no explicit mention of harms, the available evidence base for safety is limited by small sample sizes, short-term follow-up, heterogenous formulations and poor harms reporting. Systematic evaluations of CHM trials find that safety reporting is frequently incomplete with CONSORT-harms recommendations increasing risk of false reassurance. These limitations highlight the importance of systematic adverse event documentation and standardized protocols in future studies to ensure the clinical applicability of these alternative interventions.

## 5. Limitations and Future Directions

The current review has several limitations that should be acknowledged. As a narrative review, it may be subject to selection and publication bias, and only English-language studies were included, which could limit the comprehensiveness of the evidence base. In addition, the included studies vary widely in diagnostic criteria, study design, and outcome reporting, making direct comparisons difficult.

Where possible, the available evidence for each therapy was evaluated using the GRADE framework. Conventional pharmacologic agents, including letrozole, clomiphene, and gonadotropins, have high-quality evidence supporting their efficacy for ovulation induction in PCOS. In contrast, complementary therapies, such as acupuncture and CHM, generally have low-to-moderate quality evidence, primarily due to small sample sizes, heterogeneity in intervention protocols, and limited blinding or allocation concealment. Recognizing these differences clarifies the relative certainty of evidence for each therapeutic approach and highlights areas where further high-quality research is needed.

Future studies should address several methodological and conceptual gaps. First, the lack of uniform diagnostic criteria across studies hinders comparability. Future trials should therefore adopt standardized diagnostic and inclusion criteria and use objective endpoints such as ovulation rate confirmed by ultrasound or serum progesterone, as well as clinically meaningful outcomes such as pregnancy and live birth rates. Second, substantial heterogeneity exists in herbal formulations, acupuncture points, depth, and technique, treatment duration, and combination regimens. Developing and validating standardized dosing protocols and well-characterized herbal formulations would greatly improve reproducibility and allow meaningful meta-analysis. Trials directly comparing integrative approaches (e.g., letrozole combined with acupuncture or specific herbal combinations) to conventional pharmacologic therapy alone could clarify potential additive benefits and optimize individualized treatment strategies. Finally, long-term follow-up and robust safety monitoring are needed to evaluate recurrence rates, metabolic outcomes, and adverse effects. Incorporating pharmacovigilance data and post-treatment reproductive outcomes will enhance both clinical relevance and patient safety. By systematically addressing these gaps, future research can strengthen the evidence base and guide more effective, personalized integrative ovulation induction strategies for women with PCOS.

## 6. Conclusions

PCOS remains the most common cause of anovulatory infertility, and ovulation induction continues to be a cornerstone of treatment. Among pharmacologic agents, letrozole is currently considered the most effective first-line option, with clomiphene citrate and gonadotropins serving as alternatives or second-line treatments in resistant cases. Adjunctive therapies—including insulin sensitizers, dexamethasone, dopamine agonists, and emerging agents such as sildenafil, omega-3 fatty acids, and vitamin D—may further enhance outcomes when used in combination with standard agents for ovulation induction.

Complementary therapies such as acupuncture and Chinese herbal medicine (CHM) are gaining attention due to patient interest and cultural relevance. While preliminary studies suggest potential benefits for ovulation and pregnancy outcomes, current evidence remains limited and should be interpreted cautiously. At present, these therapies may be considered as adjunctive strategies in carefully selected patients, but they should not replace evidence-based pharmacologic regimens.

## Figures and Tables

**Table 1:** Randomized controlled studies on combination therapy.

10	Phase	N	Treatment	Outcomes	Side Effects
Sarkar 2024 [4]	NA	120	Letrozole + CC vs. letrozole	Similar ovulation rates: letrozole + CC combination vs. letrozole alone (71/92 (77.2%) vs. 52/83 (62.6%), RR 1.43, 95% CI 0.99–2.06)	No major adverse effects
Thabet 2024 [5]	NA	300	CC + low dosage of HCG vs. CC	Higher ovulation rate in combination therapy: CC + HCG vs. CC (26.8% vs. 6.1%)	NA
Jamal 2023 [6]	NA	136	Coenzyme Q10 + CC vs. CC	Higher ovulation rate in combination therapy: 16 (23.5%)	NA
Panda 2023 [7]	NA	80	Letrozole + CC vs. letrozole	Higher ovulation rate in combination therapy: Letrozole + CC vs. letrozole (73% vs. 38%, *p* = 0.003, RR 1.93, 95%CI 1.24–3.01)	No serious adverse events attributable to the treatments
Ji 2022 [8]	NA	60	Probiotics vs. metformin vs. probiotics + metformin combination	Higher ovulation rate in combination therapy: probiotics vs. metformin vs. combination (30% vs. 55% vs. 75%, *p* = 0.017)	NA
Rasheedy 2020 [9]	NA	186	Effect of vitamin D supplementation	Higher ovulation rate in combination therapy: treatment group vs. control group (92.5% vs. 78.5%, *p* = 0.007)	No statistically significant difference between groups regarding side effects
Mejia 2019 [10]	4	70	Letrozole + CC vs. letrozole	Higher ovulation rate in combination therapy: letrozole + CC vs. letrozole alone (77% vs. 43%)	No serious adverse events in either group
Yu 2018 [11]	NA	80	Electroacupuncture + CC vs. CC	Higher ovulation rate in combination therapy: acupuncture + CC vs. CC (86.8% vs. 64.9%, *p* < 0.05)	Medication was discontinued in 3 cases due to adverse gastrointestinal reactions
Yin 2018 [12]	NA	120	Letrozole vs. Letrozole + Chinese herbal medicine vs. Letrozole + Chinese herbal medicine + acupuncture	Higher ovulation rate in combination therapy (all *p* < 0.05)	NA
	NA				
Ma 2016 [13]	NA	90	Flying needling therapy + CC vs. flying needling therapy vs. CC	Higher ovulation rate in combination therapy: flying needling therapy + CC vs. flying needling therapy alone vs. CC alone (86.2% vs. 66.7% vs. 69.6%, *p* < 0.05)	NA
Ruan 2016 [14]	NA	170	Cold needle puncture drainage surgery alone vs. cold needle puncture drainage surgery + Bushen Quyu Recipe + acupuncture	Higher ovulation rate in combination therapy: cold needle puncture drainage surgery alone vs. cold needle puncture drainage surgery + Bushen Quyu Recipe + acupuncture (75.29% vs. 56.47%, *p* < 0.05)	NA
Wu 2016 [15]	NA	644	Berberine + letrozole vs. letrozole	Similar ovulation rates in the combination and letrozole groups (61.0% vs. 59.4%, *p* = 0.845), and significantly higher than in the berberine group (61.0% vs. 36.3%, *p* < 0.001)	Berberine was associated with a significantly higher incidence of constipation and nausea, and letrozole was associated with a significantly higher incidence of fatigue and hot flashes. No significant differences among the three groups regarding the total number of adverse events was found
El-khayat 2016 [16]	1	50	CC + metformin + pioglitazone vs. letrozole + metformin + pioglitazone	Similar ovulation rate: CC + metformin + pioglitazone vs. letrozole + metformin + pioglitazone (92.3% vs. 86.9%, *p* = 0.184)	NA
Maged 2015 [17]	NA	120	CC alone vs. CC + metformin vs. CC + N-acetylcysteine	Similar ovulation rate	NA
Li 2013 [18]	NA	100	Acupuncture + auricular point sticking vs. CC	Higher ovulation rate in acupuncture + auricular point sticking: Acupuncture + auricular point sticking vs. CC 90.0% vs. 86.0%, *p* < 0.05)	No adverse effect was observed in acupuncture group, while in CC group, varying degrees of nausea, vomiting, headache and dermatitis were observed in 29 cases
Ghanem 2013 [19]	NA	174	CC + FSH vs. FSH	Higher ovulation rate in combination therapy: CC + FSH vs. FSH (72.4% vs. 34.2%, *p* < 0.001)	NA
Ayaz 2013 [20]	NA	42	CC + metformin vs. CC	Higher ovulation rate in combination therapy: CC + metformin vs. CC alone (76.2% vs. 38.1%; *p* = 0.021)	In metformin group, 60% of the females complained of loss of appetite, and 18% had nausea and vomiting, but none discontinued therapy
Salehpour 2012 [21]	NA	180	CC + N-acetylcysteine vs. CC	Higher ovulation rate in combination therapy: CC + N-acetylcysteine vs. CC alone (45.12% vs. 28%, *p* = 0.02)	No adverse side effects and no cases of ovarian hyperstimulation syndrome were observed in the group receiving N-acetylcystein
Mohsen 2012 [22]	NA	100	Rosiglitazone + CC vs. CC	Higher ovulation rate in combination therapy: Rosiglitazone + CC vs. CC only (81.8% vs. 55.2%, *p* < 0.001)	NA
Hashim H 2010 [23]	NA	192	CC + N-acetylcysteine vs. CC + metformin	Higher ovulation rate with metformin: CC + N-acetylcysteine vs. CC + metformin (20.0% vs. 69.1%, *p* = 0.002).	NA
Zakherah 2010 [24]	NA	150	CC + tamoxifen vs. laparoscopic ovarian drilling	Similar ovulation rates (81.3% vs. 85.3%)	NA
Siebert 2009 [25]	NA	107	Metformin + CC vs. CC	Similar ovulation rates: metformin + CC vs. CC alone (65.4% vs. 65.5%, 95% CI: −18.1–18%)	NA
Ganesh 2009 [26]	NA	1387	Letrozole vs. CC + FSH vs. FSH	Higher ovulation rate in combination therapy: letrozole vs. CC + FSH vs. FSH (79.30% vs. 56.95% vs. 89.89%, *p* < 0.001)	NA
Elkind-Hirsch 2008 [27]	2	60	Metformin vs. exenatide vs. combination therapy	Higher ovulation rate in combination therapy: (*p* < 0.01)	NA
Zain 2009 [28]	NA	150	Metformin vs. CC vs. metformin + CC	Ovulation rates: metformin vs. metformin + CC (24% vs. 66.6%, OR 6.98, 95% CI 2.53–19.32), CC vs. metformin + CC (59% vs. 66.6%, *p* = 0.74)	Nausea (overall 27%), the most common side effect, was higher in COM-treated subjects than in those on monotherapy. Other reported adverse events associated with metformin were vomiting (7%) and headache (2%). Diarrhea (overall 13%) was more likely to occur with combination or metformin treatment.
Legro 2007 [29]	NA	16	Rosiglitazone or metformin vs. combination therapy	Similar ovulation rates	NA
Rouzi 2006 [30]	NA	25	Rosiglitazone + CC vs. metformin + CC	Higher ovulation rate in rosiglitazone group: rosiglitazone + CC vs. metformin + CC (64.3% vs. 36.4%, *p* = 0.035).	NA
Elnashar 2006 [31]	NA	80	CC + dexamethasone vs. CC	Higher ovulation rate in combination therapy: CC + dexamethasone vs. CC only (75% vs. 15%, *p* < 0.001)	Dexamethasone was very well tolerated, as no patients complained of any side effects
Sohrabvand 2006 [32]	NA	59	Letrozole + metformin vs. CC + metformin	Similar ovulation rates	NA
Baillargeon 2004 [33]	NA	128	Metformin vs. rosiglitazone vs. combination therapy	Higher ovulation rate in metformin and combination therapy compared to rosiglitazone group: metformin vs. rosiglitazone vs. combination therapy (93% vs. 59% vs. 90%, *p* < 0.001)	NA
Zhang 2004 [34]	NA	96	CC vs. rosiglitazone alone vs. CC + rosiglitazone	Higher ovulation rate in combination therapy: CC alone vs. rosiglitazone alone vs. CC + rosiglitazone (59% vs. 35% vs. 80%, *p* < 0.05).	NA
Parsanezhad 2002 [35]	NA	230	CC + dexamethasone vs. CC	Higher ovulation rate in combination therapy: CC + dexamethasone vs. CC (88% vs. 20%)	NA

CC, clomiphene citrate; RR, relative risk; CI, confidence interval; HCG, human chorionic gonadotropin; NA, not applicable; FSH, Follicle stimulating hormone; OR, Odds ratio.

## Data Availability

No new data were created or analyzed in this study.

## References

[1] Fauser B. (2004). Revised 2003 consensus on diagnostic criteria and long-term health risks related to polycystic ovary syndrome. Fertil. Steril..

[2] Chaudhary V., Haloi P., Munjal K., Chitme H.R. (2025). Pharmacotherapeutic importance of phytoconstituents in the management of Polycystic Ovary Syndrome (PCOS) and associated complications. Egypt. J. Basic Appl. Sci..

[3] Vyrides A.A., El Mahdi E., Giannakou K. (2022). Ovulation induction techniques in women with polycystic ovary syndrome. Front. Med..

[4] Sarkar S., Gupta N., Joseph T., Yadav B., Kunjummen A.T., Kamath M.S. (2024). Comparison of Letrozole Versus Combination Letrozole and Clomiphene Citrate (CC) for Ovulation Induction in Sub Fertile Women with Polycystic Ovarian Syndrome (PCOS)-An Open Label Randomized Control Trial. Reprod. Sci..

[5] Thabet M., Abdelhafez M.S., Elshamy M.R., Albahlol I.A., Fayala E., Wageeh A., El-Zayadi A.A., Bahgat N.A., Mohammed S.M., Mohamed A.A. (2024). Competence of Combined Low Dose of Human Chorionic Gonadotropin (HCG) and Clomiphene Citrate (CC) Versus Continued CC during Ovulation Induction in Women with CC-Resistant Polycystic Ovarian Syndrome: A Randomized Controlled Trial. Medicina.

[6] Jamal H., Waheed K., Mazhar R., Sarwar M.Z. (2023). Comparative Study of Combined Co-Enzyme Q10 And Clomiphene Citrate Vs Clomiphene Citrate Alone for Ovulation Induction In Patients with Polycystic Ovarian Syndrome. J. Pak. Med. Assoc..

[7] Panda S.R., Sharmila V., Kalidoss V.K., Hota S. (2023). A triple-blind, randomized controlled trial, comparing combined letrozole and clomiphene versus only letrozole for ovulation induction in women with polycystic ovarian syndrome. Int. J. Gynecol. Obstet..

[8] Ji X., Chen J., Xu P., Shen S., Bi Y. (2022). Effect of probiotics combined with metformin on improvement of menstrual and metabolic patterns in women with polycystic ovary syndrome: A randomized clinical trial. Gynecol. Endocrinol..

[9] Rasheedy R., Sammour H., Elkholy A., Salim Y. (2020). The efficacy of vitamin D combined with clomiphene citrate in ovulation induction in overweight women with polycystic ovary syndrome: A double blind, randomized clinical trial. Endocrine.

[10] Mejia R.B., Summers K.M., Kresowik J.D., Van Voorhis B.J. (2019). A randomized controlled trial of combination letrozole and clomiphene citrate or letrozole alone for ovulation induction in women with polycystic ovary syndrome. Fertil. Steril..

[11] Yu L., Cao L., Xie J., Shi Y. (2018). Therapeutic effects on ovulation and reproduction promotion with acupuncture and clomiphene in polycystic ovary syndrome. Zhongguo Zhen Jiu.

[12] Yin Y., Zhang Y., Zhang H., Jiang D., Guo G. (2018). Clinical therapeutic effects of acupuncture combined with Chinese herbal medicine on infertility of polycystic ovary syndrome in the patients with ovulation induction with letrozole. Zhongguo Zhen Jiu.

[13] Ma H., Quan X., Chen X., Dong Y. (2016). Flying needling therapy combined with clomiphene for ovulation failure in polycystic ovary syndrome:a randomized controlled trial. Zhongguo Zhen Jiu.

[14] Ruan H.B., Wang M.Z., Wu T.T., Liu X., Mo W.W. (2016). Clinical Effect of Bushen Quyu Recipe Combined with Acupuncture in Treatment of Clomiphene- resistant Polycystic Ovary Syndrome Infertility Patients after Cold Needle Puncture Drainage Operation. Zhongguo Zhong Xi Yi Jie He Za Zhi.

[15] Wu X.K., Wang Y.Y., Liu J.P., Liang R.N., Xue H.Y., Ma H.X., Shao X.G., Ng E.H. (2016). Randomized controlled trial of letrozole, berberine, or a combination for infertility in the polycystic ovary syndrome. Fertil. Steril..

[16] El-khayat W., Abdel Moety G., Al Mohammady M., Hamed D. (2016). A randomized controlled trial of clomifene citrate, metformin, and pioglitazone versus letrozole, metformin, and pioglitazone for clomifene-citrate-resistant polycystic ovary syndrome. Int. J. Gynecol. Obstet..

[17] Maged A.M., Elsawah H., Abdelhafez A., Bakry A., Mostafa W.A. (2015). The adjuvant effect of metformin and N-acetylcysteine to clomiphene citrate in induction of ovulation in patients with Polycystic Ovary Syndrome. Gynecol. Endocrinol..

[18] Li N. (2013). Efficacy and safety evaluation of acupuncture combined with auricular point sticking therapy in the treatment of polycystic ovary syndrome. Zhongguo Zhen Jiu.

[19] Ghanem M.E., Elboghdady L.A., Hassan M., Helal A.S., Gibreel A., Houssen M., Shaker M.E., Bahlol I., Mesbah Y. (2013). Clomiphene citrate co-treatment with low dose urinary FSH versus urinary FSH for clomiphene resistant PCOS: Randomized controlled trial. J. Assist. Reprod. Genet..

[20] Ayaz A., Alwan Y., Farooq M.U. (2013). Efficacy of combined metformin-clomiphene citrate in comparison with clomiphene citrate alone in infertile women with polycystic ovarian syndrome (PCOS). J. Med. Life.

[21] Salehpour S., Sene A.A., Saharkhiz N., Sohrabi M.R., Moghimian F. (2012). N-Acetylcysteine as an adjuvant to clomiphene citrate for successful induction of ovulation in infertile patients with polycystic ovary syndrome. J. Obstet. Gynaecol. Res..

[22] Mohsen I.A. (2012). A randomized controlled trial of the effect of rosiglitazone and clomiphene citrate versus clomiphene citrate alone in overweight/obese women with polycystic ovary syndrome. Gynecol. Endocrinol..

[23] Abu Hashim H., El Lakany N., Sherief L. (2011). Combined metformin and clomiphene citrate versus laparoscopic ovarian diathermy for ovulation induction in clomiphene-resistant women with polycystic ovary syndrome: A randomized controlled trial. J. Obstet. Gynaecol. Res..

[24] Zakherah M.S., Nasr A., El Saman A.M., Shaaban O.M., Shahin A.Y. (2010). Clomiphene citrate plus tamoxifen versus laparoscopic ovarian drilling in women with clomiphene-resistant polycystic ovary syndrome. Int. J. Gynecol. Obstet..

[25] Siebert T.I., Kruger T.F., Lombard C. (2009). Evaluating the equivalence of clomiphene citrate with and without metformin in ovulation induction in PCOS patients. J. Assist. Reprod. Genet..

[26] Ganesh A., Goswami S.K., Chattopadhyay R., Chaudhury K., Chakravarty B. (2009). Comparison of letrozole with continuous gonadotropins and clomiphene-gonadotropin combination for ovulation induction in 1387 PCOS women after clomiphene citrate failure: A randomized prospective clinical trial. J. Assist. Reprod. Genet..

[27] Elkind-Hirsch K., Marrioneaux O., Bhushan M., Vernor D., Bhushan R. (2008). Comparison of single and combined treatment with exenatide and metformin on menstrual cyclicity in overweight women with polycystic ovary syndrome. J. Clin. Endocrinol. Metab..

[28] Zain M.M., Jamaluddin R., Ibrahim A., Norman R.J. (2009). Comparison of clomiphene citrate, metformin, or the combination of both for first-line ovulation induction, achievement of pregnancy, and live birth in Asian women with polycystic ovary syndrome: A randomized controlled trial. Fertil. Steril..

[29] Legro R.S., Zaino R.J., Demers L.M., Kunselman A.R., Gnatuk C.L., Williams N.I., Dodson W.C. (2007). The effects of metformin and rosiglitazone, alone and in combination, on the ovary and endometrium in polycystic ovary syndrome. Am. J. Obstet. Gynecol..

[30] Rouzi A.A., Ardawi M.S. (2006). A randomized controlled trial of the efficacy of rosiglitazone and clomiphene citrate versus metformin and clomiphene citrate in women with clomiphene citrate-resistant polycystic ovary syndrome. Fertil. Steril..

[31] Elnashar A., Abdelmageed E., Fayed M., Sharaf M. (2006). Clomiphene citrate and dexamethazone in treatment of clomiphene citrate-resistant polycystic ovary syndrome: A prospective placebo-controlled study. Hum. Reprod..

[32] Sohrabvand F., Ansari S., Bagheri M. (2006). Efficacy of combined metformin-letrozole in comparison with metformin-clomiphene citrate in clomiphene-resistant infertile women with polycystic ovarian disease. Hum. Reprod..

[33] Baillargeon J.P., Jakubowicz D.J., Iuorno M.J., Jakubowicz S., Nestler J.E. (2004). Effects of metformin and rosiglitazone, alone and in combination, in nonobese women with polycystic ovary syndrome and normal indices of insulin sensitivity. Fertil. Steril..

[34] Zhang C.L., Gao H.Y., Zhao Z.G., Jia P. (2004). Effect of rosiglitazone on ovulation induction in women with polycystic ovary syndrome. Zhonghua Fu Chan Ke Za Zhi.

[35] Parsanezhad M.E., Alborzi S., Jahromi B.N. (2002). A prospective, double-blind, randomized, placebo-controlled clinical trial of bromocriptine in clomiphene-resistant patients with polycystic ovary syndrome and normal prolactin level. Int. J. Fertil. Womens Med..

[36] Clark J.H., Markaverich B.M. (1981). The agonistic-antagonistic properties of clomiphene: A review. Pharmacol. Ther..

[37] Hsueh A.J., Erickson G.F., Yen S.S. (1978). Sensitisation of pituitary cells to luteinising hormone releasing hormone by clomiphene citrate in vitro. Nature.

[38] Homburg R. (2005). Clomiphene citrate—End of an era? a mini-review. Hum. Reprod..

[39] Gysler M., March C.M., Mishell D.R., Bailey E.J. (1982). A decade’s experience with an individualized clomiphene treatment regimen including its effect on the postcoital test. Fertil. Steril..

[40] Wu C.H., Winkel C.A. (1989). The effect of therapy initiation day on clomiphene citrate therapy. Fertil. Steril..

[41] Hurst B.S., Hickman J.M., Matthews M.L., Usadi R.S., Marshburn P.B. (2009). Novel clomiphene “stair-step” protocol reduces time to ovulation in women with polycystic ovarian syndrome. Am. J. Obstet. Gynecol..

[42] Deveci C.D., Demir B., Sengul O., Dilbaz B., Goktolga U. (2015). Clomiphene citrate ‘stair-step’ protocol vs. traditional protocol in patients with polycystic ovary syndrome: A randomized controlled trial. Arch. Gynecol. Obstet..

[43] Agrawal K., Gainder S., Dhaliwal L.K., Suri V. (2017). Ovulation Induction Using Clomiphene Citrate Using Stair—Step Regimen versus Traditional Regimen in Polycystic Ovary Syndrome Women—A Randomized Control Trial. J. Hum. Reprod. Sci..

[44] Jones T., Ho J.R., Gualtieri M., Bruno-Gaston J., Chung K., Paulson R.J., Bendikson K.A. (2018). Clomiphene Stair-Step Protocol for Women with Polycystic Ovary Syndrome. Obstet. Gynecol..

[45] Shahgheibi S., Seyedoshohadaei F., Khezri D., Ghasemi S. (2021). Endometrial and follicular development following stair-step and traditional protocols in women with polycystic ovary syndrome: An RCT. Int. J. Reprod. Biomed..

[46] Imani B., Eijkemans M.J., te Velde E.R., Habbema J.D., Fauser B.C. (1999). Predictors of chances to conceive in ovulatory patients during clomiphene citrate induction of ovulation in normogonadotropic oligoamenorrheic infertility. J. Clin. Endocrinol. Metab..

[47] Correy J.F., Marsden D.E., Schokman F.C. (1982). The outcome of pregnancy resulting from clomiphene-induced ovulation. Aust. N. Z. J. Obstet. Gynaecol..

[48] Ahlgren M., Källén B., Rannevik G. (1976). Outcome of pregnancy after clomiphene therapy. Acta Obstet. Gynecol. Scand..

[49] Schenker J.G., Yarkoni S., Granat M. (1981). Multiple pregnancies following induction of ovulation. Fertil. Steril..

[50] Kamat A., Hinshelwood M.M., Murry B.A., Mendelson C.R. (2002). Mechanisms in tissue-specific regulation of estrogen biosynthesis in humans. Trends Endocrinol. Metab..

[51] Naftolin F., MacLusky N.J., Leranth C.Z., Sakamoto H.S., Garcia-Segura L.M. (1988). The cellular effects of estrogens on neuroendocrine tissues. J. Steroid Biochem..

[52] Mitwally M.F., Casper R.F. (2001). Use of an aromatase inhibitor for induction of ovulation in patients with an inadequate response to clomiphene citrate. Fertil. Steril..

[53] Shi L., Ye S., Gao M., Chen Y., Jin X., Zhang Z. (2022). Effect of different timing of letrozole initiation on pregnancy outcome in polycystic ovary syndrome. Front. Endocrinol..

[54] Zhu X., Fu Y. (2023). Extending letrozole treatment duration is effective in inducing ovulation in women with polycystic ovary syndrome and letrozole resistance. Fertil. Steril..

[55] He D., Jiang F. (2011). Meta-analysis of letrozole versus clomiphene citrate in polycystic ovary syndrome. Reprod. Biomed. Online.

[56] Misso M.L., Wong J.L., Teede H.J., Hart R., Rombauts L., Melder A.M., Norman R.J., Costello M.F. (2012). Aromatase inhibitors for PCOS: A systematic review and meta-analysis. Hum. Reprod. Update.

[57] Franik S., Eltrop S.M., Kremer J.A., Kiesel L., Farquhar C. (2018). Aromatase inhibitors (letrozole) for subfertile women with polycystic ovary syndrome. Cochrane Database Syst. Rev..

[58] Hu S., Yu Q., Wang Y., Wang M., Xia W., Zhu C. (2018). Letrozole versus clomiphene citrate in polycystic ovary syndrome: A meta-analysis of randomized controlled trials. Arch. Gynecol. Obstet..

[59] Wang R., Li W., Bordewijk E.M., Legro R.S., Zhang H., Wu X., Gao J., Morin-Papunen L., Homburg R., König T.E. (2019). First-line ovulation induction for polycystic ovary syndrome: An individual participant data meta-analysis. Hum. Reprod. Update.

[60] Abu-Zaid A., Gari A., Sabban H., Alshahrani M.S., Khadawardi K., Badghish E., AlSghan R., Bukhari I.A., Alyousef A., Abuzaid M. (2024). Comparison of Letrozole and Clomiphene Citrate in Pregnancy Outcomes in Patients with Polycystic Ovary Syndrome: A Systematic Review and Meta-analysis. Reprod. Sci..

[61] Salaheldin AbdelHamid A.M., Rateb A.M., Ismail Madkour W.A. (2016). Is clomiphene citrate stair-step protocol a good alternative to gonadotrophins in clomiphene-resistant PCO patients? Prospective study. J. Obstet. Gynaecol. Res..

[62] Sakar M.N., Oğlak S.C. (2021). Comparison of the efficacy of letrozole stair-step protocol with clomiphene citrate stair-step protocol in the management of clomiphene citrate-resistant polycystic ovary syndrome patients. J. Obstet. Gynaecol. Res..

[63] Al-Thuwaynee S., Swadi A.A.J. (2023). Comparing efficacy and safety of stair step protocols for clomiphene citrate and letrozole in ovulation induction for women with polycystic ovary syndrome (PCOS): A randomized controlled clinical trial. J. Med. Life.

[64] Weiss N.S., Kostova E., Nahuis M., Mol B.W.J., Van der Veen F., van Wely M. (2019). Gonadotrophins for ovulation induction in women with polycystic ovary syndrome. Cochrane Database Syst. Rev..

[65] Balen A.H., Braat D.D., West C., Patel A., Jacobs H.S. (1994). Cumulative conception and live birth rates after the treatment of anovulatory infertility: Safety and efficacy of ovulation induction in 200 patients. Hum. Reprod..

[66] Schwartz M., Jewelewicz R., Dyrenfurth I., Tropper P., Vande Wiele R.L. (1980). The use of human menopausal and chorionic gonadotropins for induction of ovulation. Sixteen years’ experience at the Sloane Hospital for Women. Am. J. Obstet. Gynecol..

[67] Fluker M.R., Urman B., Mackinnon M., Barrow S.R., Pride S.M., Yuen B.H. (1994). Exogenous gonadotropin therapy in World Health Organization groups I and II ovulatory disorders. Obstet. Gynecol..

[68] Weiss N.S., Nahuis M.J., Bordewijk E., Oosterhuis J.E., Smeenk J.M., Hoek A., Broekmans F.J., Fleischer K., de Bruin J.P., Kaaijk E.M. (2018). Gonadotrophins versus clomifene citrate with or without intrauterine insemination in women with normogonadotropic anovulation and clomifene failure (M-OVIN): A randomised, two-by-two factorial trial. Lancet.

[69] Ibrahem M.A. (2019). Simultaneous letrozole and clomiphene citrate versus letrozole alone in clomiphene citrate resistant polycystic ovary syndrome: A randomised controlled trial. Open J. Obstet. Gynecol..

[70] Sharma P., Chandra R., Sarkar A., Jindal S., Sharma A., Sharma J.C., Jaggarwal S. (2023). Assessment of Fertility Outcomes Following Combined Clomiphene and Letrozole Versus Letrozole Therapy for the Treatment of Polycystic Ovarian Syndrome Subfertility. Cureus.

[71] Du B., Ni M., Chen P. (2025). Effects of letrozole combined with clomiphene in the treatment of polycystic ovary syndrome: A meta-analysis. BMC Women’s Health.

[72] Eskandar K., Oliveira J.A., Ribeiro S.A., Chavez M.P., Zotti A.I.A., Dias Y.J.M., Novellino A.M.M. (2025). Letrozole and clomiphene versus letrozole alone for ovulation induction in women with PCOS: A systematic review and meta-analysis. Rev. Bras. Ginecol. Obstet..

[73] Ryan G.L., Moss V., Davis W.A., Sparks A., Dokras A., Van Voorhis B.J. (2005). Oral ovulation induction agents combined with low-dose gonadotropin injections and intrauterine insemination: Cost-and clinical effectiveness. J. Reprod. Med..

[74] Hajishafiha M., Dehghan M., Kiarang N., Sadegh-Asadi N., Shayegh S.N., Ghasemi-Rad M. (2013). Combined letrozole and clomiphene versus letrozole and clomiphene alone in infertile patients with polycystic ovary syndrome. Drug Des. Dev. Ther..

[75] Xia T.T., Zeng K.F., Peng Q.M. (2023). Comparison of Three Ovulation Induction Therapies for Patients with Polycystic Ovary Syndrome and Infertility. J. Clin. Pharmacol..

[76] Dai X., Li J., Fu T., Long X., Li X., Weng R., Liu Y., Zhang L. (2023). Ovulation induction using sequential letrozole/gonadotrophin in infertile women with PCOS: A randomized controlled trial. Reprod. Biomed. Online.

[77] Sharma S., Choudhary M., Swarankar V., Vaishnav V. (2021). Comparison of Tamoxifen and Clomiphene Citrate for Ovulation Induction in Women with Polycystic Ovarian Syndrome: A Prospective Study. J. Reprod. Infertil..

[78] Xuekun S.H., Siyou H.Z. (2011). Different stimulate ovulation drugs influence of early pregnancy outcome in patients with polycystic ovary syndrome. Pract. Med. J..

[79] Narayanan M., Jahaan U., Gupta N. (2019). Comparative evaluation of different cost effective ovulation induction drugs and their effect on follicular growth, endometrial thickness and pregnancy outcome. Int. J. Reprod. Contracept. Obstet. Gynecol..

[80] Sattar M.M.A., El-Halaby A.E.F., El-Shamy E.-S.A., Taha S.N. (2020). Effect of clomiphene citrate, tamoxifen, and letrozole on endometrial thickness in cycles of ovulation induction: A randomized controlled trial. Menoufia Med. J..

[81] Li Y., Yang D.-z. (2011). Letrozole, tamoxifen or clomiphene citrate for ovulation induction in women with polycystic ovarian syndrome after pretreatment: A prospective randomized trial. Chin. J. Pract. Gynecol. Obstet..

[82] Boostanfar R., Jain J.K., Mishell D.R., Paulson R.J. (2001). A prospective randomized trial comparing clomiphene citrate with tamoxifen citrate for ovulation induction. Fertil. Steril..

[83] Nardo L.G. (2004). Management of anovulatory infertility associated with polycystic ovary syndrome: Tamoxifen citrate an effective alternative compound to clomiphene citrate. Gynecol. Endocrinol..

[84] Seyedoshohadaei F., Zandvakily F., Shahgeibi S. (2012). Comparison of the effectiveness of clomiphene citrate, tamoxifen and letrozole in ovulation induction in infertility due to isolated unovulation. Iran. J. Reprod. Med..

[85] Xiang D., Chen L., Zeng L. (2016). Clinical efficacy of clomiphene and tamoxifen on infertility patients with polycystic ovary syndrome. Iran. J. Reprod. Med..

[86] Luo Q.-y. (2016). The clinical effect of letrozole, tamoxifen and clomiphene in treatment of patients with polycystic ovary syndrome infertility. Clin. Med. Res. Pract..

[87] Jie L., Li D., Yang C., Haiying Z. (2018). Tamoxifen versus clomiphene citrate for ovulation induction in infertile women. Eur. J. Obstet. Gynecol. Reprod. Biol..

[88] El-Gharib M.N., Mahfouz A.E., Farahat M.A. (2015). Comparison of letrozole versus tamoxifen effects in clomiphen citrate resistant women with polycystic ovarian syndrome. J. Reprod. Infertil..

[89] Flory J., Lipska K. (2019). Metformin in 2019. JAMA.

[90] Teede H.J., Hutchison S.K., Zoungas S. (2007). The management of insulin resistance in polycystic ovary syndrome. Trends Endocrinol. Metab..

[91] Sam S., Ehrmann D.A. (2017). Metformin therapy for the reproductive and metabolic consequences of polycystic ovary syndrome. Diabetologia.

[92] Tang T., Glanville J., Hayden C.J., White D., Barth J.H., Balen A.H. (2006). Combined lifestyle modification and metformin in obese patients with polycystic ovary syndrome. A randomized, placebo-controlled, double-blind multicentre study. Hum. Reprod..

[93] Abu Hashim H., Foda O., Ghayaty E. (2015). Combined metformin-clomiphene in clomiphene-resistant polycystic ovary syndrome: A systematic review and meta-analysis of randomized controlled trials. Acta Obstet. Gynecol. Scand..

[94] Sharpe A., Morley L.C., Tang T., Norman R.J., Balen A.H. (2019). Metformin for ovulation induction (excluding gonadotrophins) in women with polycystic ovary syndrome. Cochrane Database Syst. Rev..

[95] Lin W., Feng J., Zhou H., Chen X., Diao W., Ma P. (2022). Therapeutic efficacy of clomiphene citrate combined with metformin in patients with polycystic ovary syndrome. J. Clin. Pharm. Ther..

[96] Azargoon A., Fatemi H.M., Mirmohammadkhani M., Darzi S. (2023). Is the Co-administration of Metformin and Clomiphene Superior to Induce Ovulation in Infertile Patients with Poly Cystic Ovary Syndrome and Confirmed Insulin-Resistance: A Double Blind Randomized Clinical Trial. J. Fam. Reprod. Health.

[97] Palomba S., Falbo A., La Sala G.B. (2014). Metformin and gonadotropins for ovulation induction in patients with polycystic ovary syndrome: A systematic review with meta-analysis of randomized controlled trials. Reprod. Biol. Endocrinol..

[98] Bordewijk E.M., Nahuis M., Costello M.F., Van der Veen F., Tso L.O., Mol B.W., van Wely M. (2017). Metformin during ovulation induction with gonadotrophins followed by timed intercourse or intrauterine insemination for subfertility associated with polycystic ovary syndrome. Cochrane Database Syst. Rev..

[99] Gupta A., Jakubowicz D., Nestler J.E. (2016). Pioglitazone Therapy Increases Insulin-Stimulated Release of d-Chiro-Inositol-Containing Inositolphosphoglycan Mediator in Women with Polycystic Ovary Syndrome. Metab. Syndr. Relat. Disord..

[100] Morley L.C., Tang T., Yasmin E., Norman R.J., Balen A.H. (2017). Insulin-sensitising drugs (metformin, rosiglitazone, pioglitazone, D-chiro-inositol) for women with polycystic ovary syndrome, oligo amenorrhoea and subfertility. Cochrane Database Syst. Rev..

[101] Kim C.H., Jeon G.H., Kim S.R., Kim S.H., Chae H.D., Kang B.M. (2010). Effects of pioglitazone on ovarian stromal blood flow, ovarian stimulation, and in vitro fertilization outcome in patients with polycystic ovary syndrome. Fertil. Steril..

[102] Emekçi Özay Ö., Özay A.C., Çağlıyan E., Okyay R.E., Gülekli B. (2017). Myo-inositol administration positively effects ovulation induction and intrauterine insemination in patients with polycystic ovary syndrome: A prospective, controlled, randomized trial. Gynecol. Endocrinol..

[103] Agrawal A., Mahey R., Kachhawa G., Khadgawat R., Vanamail P., Kriplani A. (2019). Comparison of metformin plus myoinositol vs metformin alone in PCOS women undergoing ovulation induction cycles: Randomized controlled trial. Gynecol. Endocrinol..

[104] Teede H., Deeks A., Moran L. (2010). Polycystic ovary syndrome: A complex condition with psychological, reproductive and metabolic manifestations that impacts on health across the lifespan. BMC Med..

[105] Isaacs J.D., Lincoln S.R., Cowan B.D. (1997). Extended clomiphene citrate (CC) and prednisone for the treatment of chronic anovulation resistant to CC alone. Fertil. Steril..

[106] Brann D., O’Conner J., Wade M., Mahesh V. (1992). LH and FSH subunit mRNA concentrations during the progesterone-induced gonadotropin surge in ovariectomized estrogen-primed immature rats. Mol. Cell. Neurosci..

[107] Farzaneh F., Afshar F. (2020). Comparison of ovulation induction with letrozole plus dexamethasone and letrozole alone in infertile women with polycystic ovarian disease: An RCT. Int. J. Reprod. Biomed..

[108] Neblett M.F., Baumgarten S.C., Babayev S.N., Shenoy C.C. (2023). Ovulation induction with letrozole and dexamethasone in infertile patients with letrozole-resistant polycystic ovary syndrome. J. Assist. Reprod. Genet..

[109] Lobo R.A., Paul W., March C.M., Granger L., Kletzky O.A. (1982). Clomiphene and dexamethasone in women unresponsive to clomiphene alone. Obstet. Gynecol..

[110] Daly D.C., Walters C.A., Soto-Albors C.E., Tohan N., Riddick D.H. (1984). A randomized study of dexamethasone in ovulation induction with clomiphene citrate. Fertil. Steril..

[111] Trott E.A., Plouffe L., Hansen K., Hines R., Brann D.W., Mahesh V.B. (1996). Ovulation induction in clomiphene-resistant anovulatory women with normal dehydroepiandrosterone sulfate levels: Beneficial effects of the addition of dexamethasone during the follicular phase. Fertil. Steril..

[112] Parsanezhad M.E., Alborzi S., Motazedian S., Omrani G. (2002). Use of dexamethasone and clomiphene citrate in the treatment of clomiphene citrate-resistant patients with polycystic ovary syndrome and normal dehydroepiandrosterone sulfate levels: A prospective, double-blind, placebo-controlled trial. Fertil. Steril..

[113] Hernández I., Parra A., Méndez I., Cabrera V., Cravioto M.C., Mercado M., Díaz-Sánchez V., Larrea F. (2000). Hypothalamic dopaminergic tone and prolactin bioactivity in women with polycystic ovary syndrome. Arch. Med. Res..

[114] Tripathy S., Mohapatra S., M M., Chandrasekhar A. (2013). Induction of Ovulation with Clomiphene Citrate Versus Clomiphene with Bromocriptine in PCOS Patients with Normal Prolactin: A Comparative Study. J. Clin. Diagn. Res..

[115] Zahran K.M., Mostafa W.A., Abbas A.M., Khalifa M.A., Sayed G.H. (2018). Clomiphene citrate plus cabergoline versus clomiphene citrate for induction of ovulation in infertile euprolactinemic patients with polycystic ovary syndrome: A randomized clinical trial. Middle East Fertil. Soc. J..

[116] Mohammadbygi R., Yousefi S.R., Shahghaybi S., Zandi S., Sharifi K., Gharibi F. (2013). Effects of Cabergoline administration on uterine perfusion in women with polycystic ovary syndrome. Pak. J. Med. Sci..

[117] Witwit S.J. (2023). Improving pregnancy rate in infertile patients with polycystic ovarian syndrome receiving clomiphene citrate and cabergoline in euprolactinomic women in single cycle treatment. Ginekol. Pol..

[118] Csuka M.E., McCarty D.J. (1989). Aspirin and the treatment of rheumatoid arthritis. Rheum. Dis. Clin. N. Am..

[119] Jorda A., Aldasoro M., Aldasoro C., Guerra-Ojeda S., Iradi A., Vila J.M., Campos-Campos J., Valles S.L. (2020). Action of low doses of Aspirin in Inflammation and Oxidative Stress induced by aβ1-42 on Astrocytes in primary culture. Int. J. Med. Sci..

[120] Yamamoto M.M.W., de Medeiros S.F. (2019). Differential activity of the corticosteroidogenic enzymes in normal cycling women and women with polycystic ovary syndrome. Rev. Endocr. Metab. Disord..

[121] Yu Q., Wang Z., Su F., Wang M. (2021). Effectiveness and safety of aspirin combined with letrozole in the treatment of polycystic ovary syndrome: A systematic review and meta-analysis. Ann. Palliat. Med..

[122] Sereepapong W., Suwajanakorn S., Triratanachat S., Sampatanukul P., Pruksananonda K., Boonkasemsanti W., Reinprayoon D. (2000). Effects of clomiphene citrate on the endometrium of regularly cycling women. Fertil. Steril..

[123] Fetih A.N., Habib D.M., Abdelaal I.I., Hussein M., Fetih G.N., Othman E.R. (2017). Adding sildenafil vaginal gel to clomiphene citrate in infertile women with prior clomiphene citrate failure due to thin endometrium: A prospective self-controlled clinical trial. Facts Views Vis. ObGyn.

[124] Hale S.A., Jones C.W., Osol G., Schonberg A., Badger G.J., Bernstein I.M. (2010). Sildenafil increases uterine blood flow in nonpregnant nulliparous women. Reprod. Sci..

[125] Fahmy A.A., Elsokkary M., Sayed S. (2015). The Value of Oral Sildenafil in the Treatment of Female Infertility: A Randomized Clinical Trial. Life Sci. J..

[126] Ashoush S., Abdelshafy A. (2019). Sildenafil citrate adjuvant treatment in women with polycystic ovary syndrome following clomiphene failure: A randomized controlled trial. Evid. Based Women’s Health J..

[127] Mohammed W.E., Abbas M.M., Abdelazim I.A., Salman M.M. (2022). Sildenafil citrate as an adjuvant to clomiphene citrate for ovulation induction in polycystic ovary syndrome: Crossover randomized controlled trial. Prz. Menopauzalny.

[128] Li X., Luan T., Zhao C., Zhang M., Dong L., Su Y., Ling X. (2020). Effect of sildenafil citrate on treatment of infertility in women with a thin endometrium: A systematic review and meta-analysis. J. Int. Med. Res..

[129] Gutarra-Vilchez R.B., Bonfill Cosp X., Glujovsky D., Viteri-García A., Runzer-Colmenares F.M., Martinez-Zapata M.J. (2018). Vasodilators for women undergoing fertility treatment. Cochrane Database Syst. Rev..

[130] Mohamed T. (2019). Oral sildenafil for treatment of female infertility among pco patients: Randomized comparative study. Austin J. Obstet. Gynecol..

[131] Menichini D., Facchinetti F. (2020). Effects of vitamin D supplementation in women with polycystic ovary syndrome: A review. Gynecol. Endocrinol..

[132] Berridge M.J. (2018). Vitamin D deficiency: Infertility and neurodevelopmental diseases (attention deficit hyperactivity disorder, autism, and schizophrenia). Am. J. Physiol. Cell Physiol..

[133] Yang M., Shen X., Lu D., Peng J., Zhou S., Xu L., Zhang J. (2023). Effects of vitamin D supplementation on ovulation and pregnancy in women with polycystic ovary syndrome: A systematic review and meta-analysis. Front. Endocrinol..

[134] Tóth B.E., Takács I., Valkusz Z., Jakab A., Fülöp Z., Kádár K., Putz Z., Kósa J.P., Lakatos P. (2025). Effects of Vitamin D3 Treatment on Polycystic Ovary Symptoms: A Prospective Double-Blind Two-Phase Randomized Controlled Clinical Trial. Nutrients.

[135] Ko J.K.Y., Yung S.S.F., Lai S.F., Wan R.S.F., Wong C.K.Y., Wong K., Cheung C.L., Ng E.H.Y., Li R.H.W. (2024). Effect of vitamin D in addition to letrozole on the ovulation rate of women with polycystic ovary syndrome: Protocol of a multicentre randomised double-blind controlled trial. BMJ Open.

[136] Hammiche F., Vujkovic M., Wijburg W., de Vries J.H., Macklon N.S., Laven J.S., Steegers-Theunissen R.P. (2011). Increased preconception omega-3 polyunsaturated fatty acid intake improves embryo morphology. Fertil. Steril..

[137] Wathes D.C., Abayasekara D.R., Aitken R.J. (2007). Polyunsaturated fatty acids in male and female reproduction. Biol. Reprod..

[138] Thomas M., Bao B., Williams G. (1997). Dietary fats varying in their fatty acid composition differentially influence follicular growth in cows fed isoenergetic diets. J. Anim. Sci..

[139] Wise L.A., Wesselink A.K., Tucker K.L., Saklani S., Mikkelsen E.M., Cueto H., Riis A.H., Trolle E., McKinnon C.J., Hahn K.A. (2018). Dietary fat intake and fecundability in 2 preconception cohort studies. Am. J. Epidemiol..

[140] Nadjarzadeh A., Dehghani-Firouzabadi R., Daneshbodi H., Lotfi M.H., Vaziri N., Mozaffari-Khosravi H. (2015). Effect of omega-3 supplementation on visfatin, adiponectin, and anthropometric indices in women with polycystic ovarian syndrome. J. Reprod. Infertil..

[141] Phelan N., O’Connor A., Kyaw Tun T., Correia N., Boran G., Roche H.M., Gibney J. (2011). Hormonal and metabolic effects of polyunsaturated fatty acids in young women with polycystic ovary syndrome: Results from a cross-sectional analysis and a randomized, placebo-controlled, crossover trial. Am. J. Clin. Nutr..

[142] Trop-Steinberg S., Heifetz E.M., Azar Y., Kafka I., Weintraub A., Gal M. (2023). Omega-3 Intake Improves Clinical Pregnancy Rate in Polycystic Ovary Syndrome Patients: A Double-Blind, Randomized Study. Isr. Med. Assoc. J..

[143] Izhar R., Husain S., Tahir M.A., Husain S. (2022). Effect of Administrating Coenzyme Q10 with Clomiphene Citrate on Ovulation Induction in Polycystic Ovary Syndrome Cases with Resistance to Clomiphene Citrate: A Randomized Controlled Trial. J. Reprod. Infertil..

[144] Wang Y., Bao H., Cong J., Qu Q. (2025). Comparative effects of acupuncture and metformin on insulin sensitivity in women with polycystic ovary syndrome: A systematic review and meta-analysis. Front. Endocrinol..

[145] Ye Y., Zhou C.C., Hu H.Q., Fukuzawa I., Zhang H.L. (2022). Underlying mechanisms of acupuncture therapy on polycystic ovary syndrome: Evidences from animal and clinical studies. Front. Endocrinol..

[146] Yang H., Xiao Z.Y., Yin Z.H., Yu Z., Liu J.J., Xiao Y.Q., Zhou Y., Li J., Yang J., Liang F.R. (2023). Efficacy and safety of acupuncture for polycystic ovary syndrome: An overview of systematic reviews. J. Integr. Med..

[147] Li Y., Zhang L., Gao J., Yan J., Feng X., He X., Jin H., Li X., Cui Z., Zhao J. (2021). Effect of Acupuncture on Polycystic Ovary Syndrome in Animal Models: A Systematic Review. Evid. Based Complement. Altern. Med..

[148] Zhao Q.Y., Sun Y., Zhou J., Gao Y.L., Ma G.Z., Hu Z.H., Wang Y., Shi Y. (2021). Effectiveness of herb-partitioned moxibustion combined with electroacupuncture on polycystic ovary syndrome in patients with symptom pattern of kidney deficiency and phlegm-dampne. J. Tradit. Chin. Med..

[149] Chen X., Tang H., Liang Y., Wu P., Xie L., Ding Y., Yang P., Long B., Lin J. (2021). Acupuncture regulates the autophagy of ovarian granulosa cells in polycystic ovarian syndrome ovulation disorder by inhibiting the PI3K/AKT/mTOR pathway through LncMEG3. Biomed. Pharmacother..

[150] Song Y.J., Liang F.X., Wu S., Yang H.S., Chen L., Huang Q., Tang H.T., Lu W., Wang H., Chen S. (2019). Network meta-analysis on the effects of the acupuncture-related therapy on ovulation rate and pregnancy rate in patients with polycystic ovary syndrome. Zhongguo Zhen Jiu.

[151] Wu J., Chen D., Liu N. (2020). Effectiveness of acupuncture in polycystic ovary syndrome: A systematic review and meta-analysis of randomized controlled trials. Medicine.

[152] Yang L., Yang W., Sun M., Luo L., Li H.R., Miao R., Pang L., Chen Y., Zou K. (2023). Meta analysis of ovulation induction effect and pregnancy outcome of acupuncture & moxibustion combined with clomiphene in patients with polycystic ovary syndrome. Front. Endocrinol..

[153] Chen X., Lan Y., Yang L., Liu Y., Li H., Zhu X., Zhao Y., Long C., Wang M., Xie Q. (2022). Acupuncture combined with metformin versus metformin alone to improve pregnancy rate in polycystic ovary syndrome: A systematic review and meta-analysis. Front. Endocrinol..

[154] Lim C.E., Ng R.W., Xu K., Cheng N.C., Xue C.C., Liu J.P., Chen N. (2016). Acupuncture for polycystic ovarian syndrome. Cochrane Database Syst. Rev..

[155] Jo J., Lee Y.J., Lee H. (2017). Acupuncture for polycystic ovarian syndrome: A systematic review and meta-analysis. Medicine.

[156] de Oliveira N.M., Machado J., Lopes L., Criado M.B. (2023). A Review on Acupuncture Efficiency in Human Polycystic Ovary/Ovarian Syndrome. J. Pharmacopunct..

[157] Wu Y., Xiao Q., Wang S., Xu H., Fang Y. (2024). Effectiveness of Acupuncture for Infertility in Patients with Polycystic Ovary Syndrome: A Systematic Review and Network Meta-Analysis. Endocr. Metab. Immune Disord. Drug Targets.

[158] Bai T., Deng X., Bi J., Ni L., Li Z., Zhuo X. (2024). The effects of acupuncture on patients with premature ovarian insufficiency and polycystic ovary syndrome: An umbrella review of systematic reviews and meta-analyses. Front. Med..

[159] Huang S., Hu M., Ng E.H.Y., Stener-Victorin E., Zheng Y., Wen Q., Wang C., Lai M., Li J., Gao X. (2020). A multicenter randomized trial of personalized acupuncture, fixed acupuncture, letrozole, and placebo letrozole on live birth in infertile women with polycystic ovary syndrome. Trials.

[160] Deng Y.P., Zhou Y.L., Wei T.T., He G.S., Zhu Z.X., Zhang S.N., Liu M.J., Xue J.J., Zhang W.X., Yang X.G. (2024). Combined traditional Chinese medicine therapy for the treatment of infertility with polycystic ovary syndrome: A network meta-analysis of randomized controlled trials. Medicine.

[161] Li P., Peng J., Ding Z., Zhou X., Liang R. (2022). Effects of Acupuncture Combined with Moxibustion on Reproductive and Metabolic Outcomes in Patients with Polycystic Ovary Syndrome: A Systematic Review and Meta-Analysis. Evid. Based Complement. Altern. Med..

[162] Hung S.W., Li Y., Chen X., Chu K.O., Zhao Y., Liu Y., Guo X., Man G.C., Wang C.C. (2022). Green Tea Epigallocatechin-3-Gallate Regulates Autophagy in Male and Female Reproductive Cancer. Front. Pharmacol..

[163] Suhail M., Rehan M., Tarique M., Tabrez S., Husain A., Zughaibi T.A. (2022). Targeting a transcription factor NF-κB by green tea catechins using in silico and in vitro studies in pancreatic cancer. Front. Nutr..

[164] Cai B., Wang Q., Zhong L., Liu F., Wang X., Chen T. (2024). Integrating Network Pharmacology, Transcriptomics to Reveal Neuroprotective of Curcumin Activate PI3K / AKT Pathway in Parkinson’s Disease. Drug Des. Dev. Ther..

[165] Buhrmann C., Mobasheri A., Busch F., Aldinger C., Stahlmann R., Montaseri A., Shakibaei M. (2011). Curcumin modulates nuclear factor kappaB (NF-kappaB)-mediated inflammation in human tenocytes in vitro: Role of the phosphatidylinositol 3-kinase/Akt pathway. J. Biol. Chem..

[166] Wu S.K., Wang L., Wang F., Zhang J. (2024). Resveratrol improved mitochondrial biogenesis by activating SIRT1/PGC-1α signal pathway in SAP. Sci. Rep..

[167] Hassanein E.H.M., Althagafy H.S., Baraka M.A., Abd-Alhameed E.K., Ibrahim I.M., Abd El-Maksoud M.S., Mohamed N.M., Ross S.A. (2024). The promising antioxidant effects of lignans: Nrf2 activation comes into view. Naunyn Schmiedebergs Arch. Pharmacol..

[168] Shen W., Jin B., Pan Y., Han Y., You T., Zhang Z., Qu Y., Liu S., Zhang Y. (2021). The Effects of Traditional Chinese Medicine-Associated Complementary and Alternative Medicine on Women with Polycystic Ovary Syndrome. Evid. Based Complement. Altern. Med..

[169] Zhang J., Li T., Zhou L., Tang L., Xu L., Wu T., Lim D.C. (2010). Chinese herbal medicine for subfertile women with polycystic ovarian syndrome. Cochrane Database Syst. Rev..

[170] Kwon C.Y., Lee B., Park K.S. (2018). Oriental herbal medicine and moxibustion for polycystic ovary syndrome: A meta-analysis. Medicine.

[171] Arentz S., Smith C.A., Abbott J., Bensoussan A. (2017). Nutritional supplements and herbal medicines for women with polycystic ovary syndrome; a systematic review and meta-analysis. BMC Complement. Altern. Med..

[172] Wang L., Liang R., Tang Q., Zhu L. (2021). An Overview of Systematic Reviews of Using Chinese Medicine to Treat Polycystic Ovary Syndrome. Evid. Based Complement. Altern. Med..

[173] Lin Y., Zeng H., Lin J., Peng Y., Que X., Wang L., Chen L., Bai N. (2024). Evaluating the therapeutic potential of moxibustion on polycystic ovary syndrome: A rat model study on gut microbiota and metabolite interaction. Front. Cell Infect. Microbiol..

[174] Mazzei R., Patitucci A., Magariello A., Russo G., Tagarelli G. (2025). From Hippocrates to Italian traditional medicine: Ethnopharmacological evidence for a potential pharmacological perspective in the management of polycystic ovary syndrome. J. Ethnopharmacol..

[175] Khodaeifar F., Bagher F.S.M., Khaki A., Torbati M., Olad S.M.E., Khaki A.A., Shokoohi M., Dalili A.H. (2019). Investigating the role of hydroalcoholic extract of Apium graveolens and Cinnamon zeylanicum on metabolically change and ovarian oxidative injury in a rat model of polycystic ovary syndrome. Int. J. Women’s Health Reprod. Sci..

[176] Kian E.M., Barancheshmeh M., Najafzadehvarzi H., Ghoreishi S.M., Shokrzadeh N. (2025). Therapeutic potential of fennel essential oil and manganese in modulating steroidal hormonal imbalance and ovarian alterations in rats with polycystic ovarian syndrome: An experimental study. Int. J. Reprod. Biomed..

[177] Mahood R.A.-H. (2012). Effects of Pimpinella anisum oil extract on some biochemical parameters in mice experimentally induced for human polycystic ovary syndrome. J. Biotechnol. Res. Cent..

[178] Li J., Long H., Cong Y., Gao H., Lyu Q., Yu S., Kuang Y. (2021). Quercetin prevents primordial follicle loss via suppression of PI3K/Akt/Foxo3a pathway activation in cyclophosphamide-treated mice. Reprod. Biol. Endocrinol..

[179] Shah M., Shrivastva V.K., Mir M.A., Sheikh W.M., Ganie M.A., Rather G.A., Shafi M., Bashir S.M., Ansari M.A., Al-Jafary M.A. (2023). Effect of quercetin on steroidogenesis and folliculogenesis in ovary of mice with experimentally-induced polycystic ovarian syndrome. Front. Endocrinol..

[180] Wal A., Wal P., Saraswat N., Wadhwa S. (2021). A detailed review on herbal treatments for treatment of PCOS-Polycystic ovary syndrome (PCOS). Curr. Nutraceuticals.

[181] Heshmati J., Moini A., Sepidarkish M., Morvaridzadeh M., Salehi M., Palmowski A., Mojtahedi M.F., Shidfar F. (2021). Effects of curcumin supplementation on blood glucose, insulin resistance and androgens in patients with polycystic ovary syndrome: A randomized double-blind placebo-controlled clinical trial. Phytomedicine.

[182] Yu J., Ding C., Hua Z., Jiang X., Wang C. (2021). Protective effects of berberine in a rat model of polycystic ovary syndrome mediated via the PI3K/AKT pathway. J. Obstet. Gynaecol. Res..

[183] Peng F., Hu Y., Peng S., Zeng N., Shi L. (2022). Apigenin exerts protective effect and restores ovarian function in dehydroepiandrosterone induced polycystic ovary syndrome rats: A biochemical and histological analysis. Ann. Med..

[184] Darabi P., Khazali H., Mehrabani Natanzi M. (2020). Therapeutic potentials of the natural plant flavonoid apigenin in polycystic ovary syndrome in rat model: Via modulation of pro-inflammatory cytokines and antioxidant activity. Gynecol. Endocrinol..

[185] Mansour A., Samadi M., Sanginabadi M., Gerami H., Karimi S., Hosseini S., Shirzad N., Hekmatdoost A., Mahdavi-Gorabi A., Mohajeri-Tehrani M.R. (2021). Effect of resveratrol on menstrual cyclicity, hyperandrogenism and metabolic profile in women with PCOS. Clin. Nutr..

[186] Luo M., Zheng L.W., Wang Y.S., Huang J.C., Yang Z.Q., Yue Z.P., Guo B. (2021). Genistein exhibits therapeutic potential for PCOS mice via the ER-Nrf2-Foxo1-ROS pathway. Food Funct..

[187] Pavithra L., Ilango K. (2023). Identification of phytoconstituents for combating Polycystic ovarian syndrome through in silico techniques. Indian J. Biochem. Biophys..

[188] Khanage S.G., Subhash T.Y., Bhaiyyasaheb I.R. (2019). Herbal drugs for the treatment of polycystic ovary syndrome (PCOS) and its complications. Pharm. Res..

[189] Zhou X., Ma Q., Yan Z., Wang Y., Qin J., Tong T., Liang R., Li Y., Wang Y., Chen J. (2023). Efficacy and safety of Chinese patent medicine Xiao Yao San in polycystic ovary syndrome: A systematic review and meta-analysis. J. Ethnopharmacol..

[190] Rong A., Ta N., E L., Meng W. (2022). Add-on effect of the Guizhi Fuling formula for management of reduced fertility potential in women with polycystic ovary syndrome: A systematic review and meta-analysis of randomized controlled trials. Front. Endocrinol..

[191] Tang X., Huang Q., Wang C., Zhang D., Dong S., Yu C. (2021). Kuntai Capsule Combined with Letrozole on Gonadal Hormone Levels and Ovarian Function in Patients with PCOS: A Systematic Review and Meta-Analysis. Front. Endocrinol..

[192] Ma Q.W., Tan Y. (2017). Effectiveness of co-treatment with traditional Chinese medicine and letrozole for polycystic ovary syndrome: A meta-analysis. J. Integr. Med..

[193] Ried K. (2015). Chinese herbal medicine for female infertility: An updated meta-analysis. Complement. Ther. Med..

[194] Sha Y., Zhu J., Shen F. (2024). A meta-analysis of efficacy on dexamethasone and clomiphene in the treatment of polycystic ovary syndrome patients. BMC Women’s Health.

[195] Lai L., Flower A., Prescott P., Wing T., Moore M., Lewith G. (2017). Standardised versus individualised multiherb Chinese herbal medicine for oligomenorrhoea and amenorrhoea in polycystic ovary syndrome: A randomised feasibility and pilot study in the UK. BMJ Open.

[196] Arentz S., Smith C.A., Abbott J., Fahey P., Cheema B.S., Bensoussan A. (2017). Combined Lifestyle and Herbal Medicine in Overweight Women with Polycystic Ovary Syndrome (PCOS): A Randomized Controlled Trial. Phytother. Res..

[197] Malik S., Saeed S., Saleem A., Khan M.I., Khan A., Akhtar M.F. (2023). Alternative treatment of polycystic ovary syndrome: Pre-clinical and clinical basis for using plant-based drugs. Front. Endocrinol..

[198] McKennon S.A., Feingold K.R., Ahmed S.F., Anawalt B., Blackman M.R., Boyce A., Chrousos G., Corpas E., de Herder W.W., Dhatariya K., Dungan K. (2000). Non-Pharmaceutical Intervention Options for Type 2 Diabetes: Complementary & Integrative Health Approaches (Including Natural Products And Mind/Body Practices). Endotext.

[199] Brancheau D., Patel B., Zughaib M. (2015). Do cinnamon supplements cause acute hepatitis?. Am. J. Case Rep..

[200] Liao C.C., Lin K.T. (2024). Pseudo-Hyperaldosteronism Arising from Licorice Cough Syrup Self-Ingestion: A Case Report. Reports.

